# Mapping population mental health concerns related to COVID-19 and the consequences of physical distancing: a Google trends analysis

**DOI:** 10.12688/wellcomeopenres.15870.2

**Published:** 2020-06-10

**Authors:** Duleeka Knipe, Hannah Evans, Amanda Marchant, David Gunnell, Ann John

**Affiliations:** 1 Population Health Sciences, University of Bristol, Bristol, UK; 2 Population Data Sciences, Swansea University, Swansea, UK; 3 National Institute of Health Research Biomedical Research Centre, University Hospitals Bristol NHS Foundation Trust, Bristol, UK

**Keywords:** COVID-19, Suicide, Mental health, Pandemic, Economic, Depression, Anxiety, Coronavirus

## Abstract

**Background:** The 2020 Coronavirus pandemic is a major international public health challenge.  Governments have taken public health protection measures to reduce the spread of the virus through non-pharmalogical measures. The impact of the pandemic and the public health response on individual and population mental health is unknown.

**Methods:** We used Google Trends data (1 Jan 2020 -  30 Mar 2020) to investigate the impact of the pandemic and government measures to curb it on people’s concerns, as indexed by changes in search frequency for topics indicating mental distress, social and economic stressors and mental health treatment-seeking. We explored the changes of key topics in Google trends in Italy, Spain, USA, UK, and Worldwide in relation to sentinel events during the pandemic.

**Results:** Globally there appears to be significant concerns over the financial and work-related consequences of the pandemic, with some evidence that levels of fear are rising. Conversely relative searching for topics related to depression and suicide fell after the pandemic was announced, with some evidence that searches for the latter have risen recently. Concerns over education and access to medication appear to be particular social stressors. Whilst searches for face-to-face treatments have declined, those for self-care have risen.

**Conclusions:** Monitoring Google trends shows promise as a means of tracking changing public concerns. In weeks to come it may enable policy makers to assess the impact of their interventions including those aiming to limit negative consequences, such as government funded financial safety nets.

The 2020 COVID-19 pandemic is the largest global public health challenge of this century, with over 200,000 deaths and almost three million people infected ^1^. In the absence of effective vaccines or treatments, governments worldwide are trying to contain the disease using public health measures including physical distancing and self-isolation. There are concerns that the pandemic and the public health response may adversely affect population mental health e.g. through job loss, debt and social isolation ^2, 3^. However, the general public’s reactions to this crisis and the impacts of these measures are unknown. A better understanding of concerns during this emergency may help us respond better to community need.

Google Trends is a publicly available data source of real-time internet search patterns, and has been used previously for health surveillance and research ^4, 5^. We aimed to use Google trends data to investigate the impact of the pandemic on peoples’ concerns and mental health, as indexed by changes in search frequency for terms indicating mental distress, social and economic stressors arising as a result of government measures to curb the epidemic and treatment seeking.

## Methods

### Data source

We used Google Trends for this analysis. We used the checklist previously suggested for documentation and development of our Google Trends analysis ^6^.

Google does not provide information on absolute numbers of searches. Instead it provides an indexed value to display search activity for a given term/topic at a specific period, time and area. This value is scaled from 0 to 100 with 100 representing the maximum searching activity for a particular term/topic in a given time period/area with search volumes for days/weeks/months given relative to this. Periods with very low search volumes are identified as zero activity

We identified key dates in relation to the pandemic from a number of sources ( Table 1). The date of the first death in each country was identified through news articles and as such are dates of first report, rather than date the death occurred. The date of first 10 deaths for each country was sourced from the World Health Organisation (WHO) daily situation reports. These dates may be subject to change where COVID-19 is identified post-mortem. The government enforced lockdown dates were also identified through news articles. In the USA where different states govern and enforce rules independently, the date for the first state-wide lock down was used, in this instance for California. WHO declared a global pandemic on 12 March 2020.

**Table 1.** Dates used in analysis of Google Trends.

	Worldwide	Italy	Spain	UK	USA
Date of first death	09/01/2020 ^7^	21/02/2020 ^8^	13/02/2020 ^9^	05/03/2020 ^10^	28/02/2020 ^11^
First 10 deaths	22/01/2020 ^12^	26/02/2020 ^13^	09/03/2020 ^14^	14/03/2020 ^15^	06/03/2020 ^16^
First lockdown		09/03/2020 ^17^	14/03/2020 ^18^	23/03/2020 ^19^	19/03/2020 ^20^

### Search strategy

Searches were carried out on 2 Apr 2020 to include all data available on that day (data available up until 30 Mar 2020) and included all query categories. We grouped the searches into four main themes: levels of mental distress, social stressors, economic stressors and treatment seeking. These were examined in Italy, Spain, USA, UK, and Worldwide. The countries were selected to represent locations with the largest numbers of COVID-19 deaths (Italy, Spain) or those predicted to experience large numbers of deaths but which were, at the time of analysis, earlier on the epidemic curve to investigate whether events occurring elsewhere in the world impacted on local concerns (UK, USA). We examined searches for the period between 1 Jan 2020 and 30 Mar 2020 to assess the immediate impact of the pandemic on population concerns. Google provides data during this period by day (90 data points for each country). In addition to this, we examined searches for the period 1 Jan 2019–30 Mar 2020 to assess whether any seasonal patterns in searching accounted for any of the changes seen in Jan–March 2020. Google provides search data summarised by week for this time period (65 data points for each country).

We explored the changes in trends of key topics in Google Trends. Topics are a group of related terms that share the same concept in any language. The use of topics in Google Trends negates the need for translating search terms. The chosen topics are outlined in Table 2 grouped into general themes. To indicate what search terms would be included in each topic, we looked at the top related search terms for each included topic ( Table 3). It is important to note that for some topics there are overlapping related search terms (e.g. the topics depression and anxiety both include the “anxiety” search term). Google trends allows a maximum of 5 topics to be displayed in a single graph. We selected five topics which showed stability of trends (as a marker of frequency) and which provided a meaningful addition to the broad themes we were exploring. We identified topics to be included in this analysis based on mental distress markers which we hypothesised would be associated with the pandemic, and stressors which were being discussed in the media, Government and WHO policy briefings, and preliminary review of concerns in relation to suicide ^2^ at the time of this analysis (March 2020). We also drew upon the experiences of the lead author (DK) who was acting as a UK community support volunteer during the outbreak, to select topics to explore under the themes of economic and social stressors. The topics explored under the treatment seeking theme were selected based on popular methods of treatment seeking. Previous analyses have highlighted that different data are provided for search terms entered with and without quotation marks in Google Trends ^6, 21^. As we are exploring search topics in Google Trends, as opposed to terms, the use of quotation marks is not applicable. A list of topics explored but not included are also summarised in Table 2 – the topics excluded either had very low indexed search values (i.e. close to 0) or were unstable over time. The simultaneous download of multiple topics allows for comparison of terms relative to each other.

**Table 2.** General themes and topic areas searched.

	Mental distress	Economic Stressors	Social stressors	Treatment seeking
Included in main search	Depression	Eviction	Pharmacy	Cognitive behavioural therapy
	Anxiety	Mortgage loan	Education	Self-care
	Suicide	Unemployment	Abuse	Counselling
	Fear	Food bank	Alcohol drink	Crisis hotline
	Loneliness	Welfare	Divorce	Mindfulness
Topics explored but not included	Suicidal ideation	Job	Argument	Self-treatment
	Stress	Debt	Domestic abuse	Self-help
	Mental health	Universal credit	Substance misuse	Meditation
		Benefits	Legal separation	
		Bills	Neglect	
		Job search	Social care	
		Recession	Child abuse	
			Domestic violence	
			Home schooling	
			Alcohol	

**Table 3.** Topics included and the top 3 related searches in Google.

Included Topic	Related search terms *
Depression	Depression, depressed, anxiety
Anxiety	Anxiety, anxious, angst
Suicide	Suicide, suicidal, commit suicide
Fear	Fear, scared, afraid
Loneliness	Lonely, loneliness, lonely song
Eviction	Eviction, evicted, evict
Mortgage loan	Mortgage, mortgage calculator, home loan
Unemployment	Welfare, social welfare, payment
Food bank	Food bank, pantry, food pantry
Welfare	Unemployment, unemployment insurance, unemployment rate
Pharmacy	Pharmacy, Pharmacy near me, all-night drugstore
Education	Education, school education, child education
Abuse	Abuse, child abuse, abusive
Alcohol drink	Alcohol, liquor, alcoholic
Divorce	Divorce, divorced, how to divorce
Cognitive behavioural therapy	CBT, cognitive, cognitive therapy
Self-care	Self care, self care quotes, how to self care
Counselling	Counselling, counsellor, counselling guidance
Crisis hotline	Hotline, suicide, suicide hotline
Mindfulness	Mindfulness, mindful, meditation

*Based on worldwide searches regardless of language and spelling errors were ignored. CBT – cognitive behavioural therapy

Previous analysis of Google Trends data has highlighted that slightly different relative search values are provided by Google for the same search (with the same parameters) on different days ^21^. This has been highlighted to be a problem when data for earlier years are downloaded and for smaller countries. As a sensitivity analysis we generated an averaged dataset for the topics included in this analysis. The averaged dataset was created by re-running the same Google Trends search query with the same parameters for time period (i.e. 1 Jan 2020 – 30 Mar 2020), location (e.g. USA), and each set of topics (e.g. mental distress topics) on 7 different days. We took the average value for each search topic (20 topics) on each of the 90 data points for all 5 settings, as it was recorded on each of the 7 separate days. We then correlated that 7-day average with values for the 90 days (1 Jan 2020–30 Mar 2020) used in the main analysis (which used values as they stood when the data were extracted on 2 Apr 2020). We checked to see whether the use of the averaged dataset would have altered our overall conclusions.

### Data management and analysis

We provide graphical presentations of Google Trends data by country and themes explored for each time period (1 Jan 2019 – 30 Mar 2020, and 1 Jan 2020 – 1 Mar 2020). Key dates related to the pandemic are marked on graphs for the 1 Jan 2020 – 30 Mar 2020 period. In addition to the sensitivity analysis outlined in the previous section , we also removed the single topic that dominated some themes to investigate the remaining topics. STATA 16 was used to manage data and for creating graphs.

## Results

We explored Google trends data for two time periods for each of the selected countries and worldwide for the selected themes. Trends in searches for specific topics worldwide and by country for 1 Jan 2019 to 30 Mar 2020 showed no evidence to suggest that changes during the current pandemic are similar to trends observed at the same time in the previous year (data not shown but available as underlying data ^22^).

**Figure 1.**
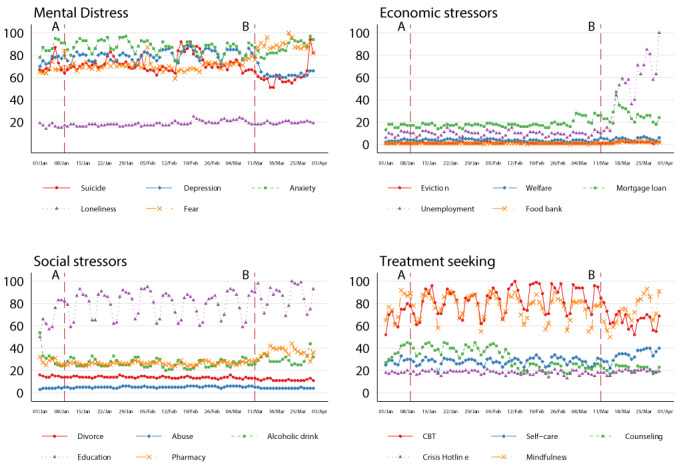
Trends in relative search volumes* of key topics worldwide between 1 Jan 2020 and 30 Mar 2020. A: Date of first Covid-19 related death; B: Date Covid-19 declared a global pandemic by WHO; *: Search activity displayed as a scaled value between 0 and 100 with 100 indicating the maximum searching for a given topic(s) over a given time period. The brief rise in suicide-searching mid-Feb coincides with the suicide of a celebrity (Caroline Flack, 15thFeb).

Focusing on trends between 1 Jan 2020 to 30 Mar 2020, there appears to be growing world-wide concerns over the financial and work-related consequences of the pandemic, with some evidence that levels of fear are rising ( Figure 1). Conversely searching for topics related to depression and suicide fell after the pandemic was declared, with some evidence that searches for the latter have risen recently. Concerns over education and access to medication (pharmacy, Figure 1) appear to be particular social stressors. Whilst searches for face-to-face treatments (i.e. cognitive behavioural therapy (CBT), counselling) have declined, those for self-care have risen. We observed a “sawtooth pattern” in trends reflecting weekday versus weekend searching.

**Figure 2.**
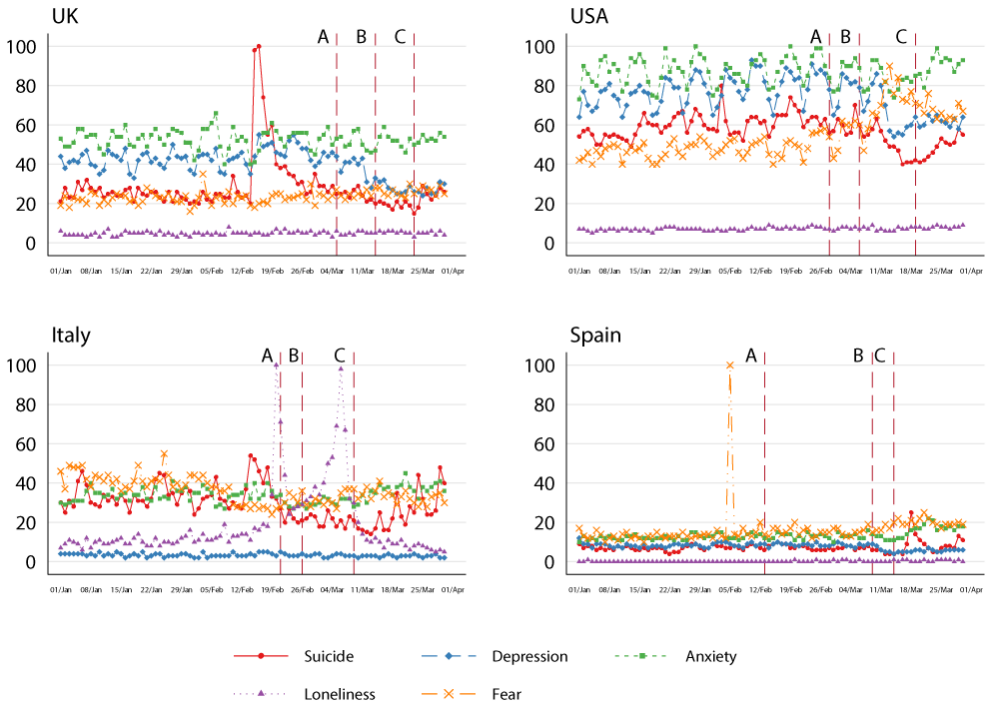
Trends in relative search volumes of key topics indicating mental distress in the UK, USA, Italy and Spain between 1 Jan 2020 and 30 Mar 2020. Showing figures for daily searches. A: Date of first Covid-19 related death; B: Date of first 10 deaths; C: Date of lockdown.

In the UK, USA, and Italy there is an indication that suicide related searches started to fall as the number of COVID-19 deaths started to rise, with searches starting to increase again after the lockdown was announced in each country ( Figure 2). In the UK the spike in suicide related Google searches in February 2020 coincided with the suicide death of TV presenter Caroline Flack (February 15 ^th^ 2020). Similar falls and rises in depression and loneliness-related Google searches were observed in the UK and Italy (respectively), whereas in other countries searches for the topic of depression and loneliness remained stable throughout the period. Levels of anxiety (as indicated by searches) were stable in all countries, with the exception of Spain, where anxiety related searches started to rise after lockdown. In the USA, levels of fear started to rise as the number of COVID-19 deaths increased.

**Figure 3.**
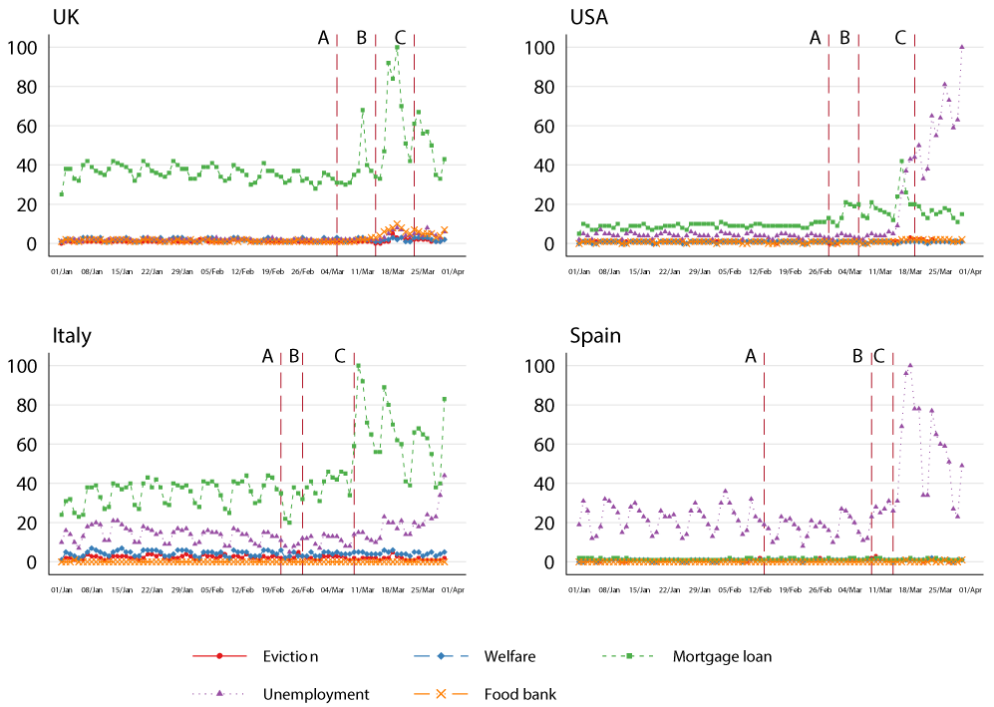
Trends in relative search volumes of key topics indicating economic stressors in the UK, USA, Italy and Spain between 1 Jan 2020 and 30 Mar 2020. Showing figures for daily searches. A: Date of first Covid-19 related death; B: Date of first 10 deaths; C: Date of lockdown.

Trends in searching for terms indicating economic concerns are shown in Figure 3. There was a marked increase in searches for mortgage loans towards the latter end of the time series in the UK, Italy and USA, which coincides with the rising number of COVID-19 related deaths. It is noteworthy that these rises in the UK and USA pre-dated lockdown, indicating people were predicting this might happen or were feeling the impact of behavioural changes on their businesses (e.g. fewer customers in restaurants / cafes). Unemployment-related searches rose sharply just before lockdown in the USA and after lockdown in Italy and Spain. In the UK there is an indication that searches related to unemployment and food banks started to rise as the number of COVID-19 deaths started to increase.

**Figure 4.**
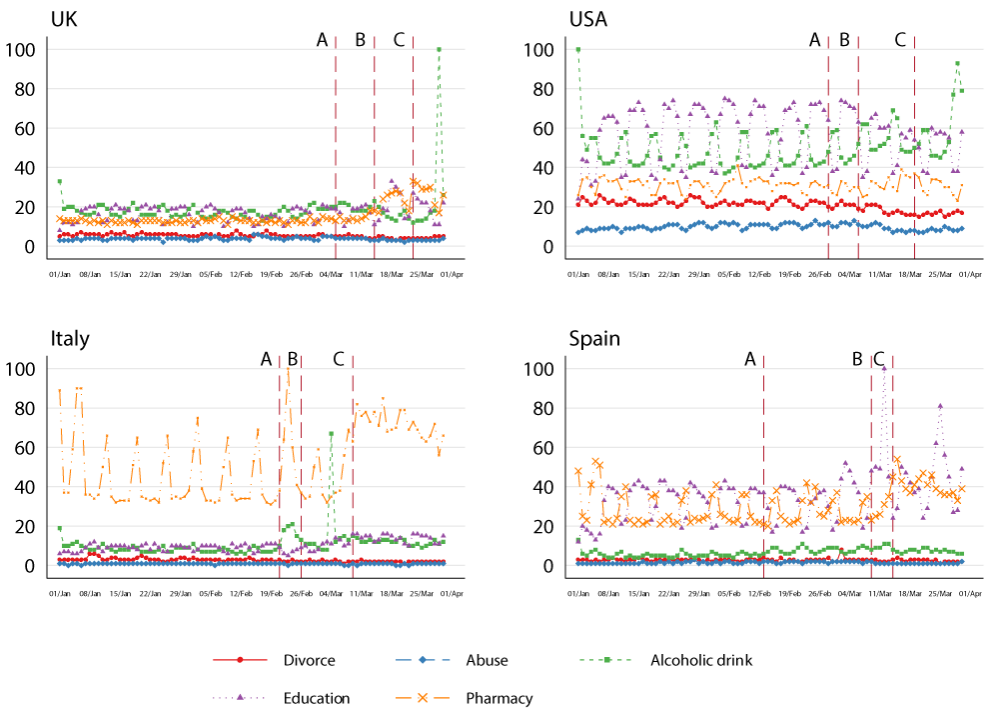
Trends in relative search volumes of key topics indicating social stressors in the UK, USA, Italy and Spain between 1 Jan 2020 and 30 Mar 2020. Showing figures for daily searches. A: Date of first Covid-19 related death; B: Date of first 10 deaths; C: Date of lockdown.

The specific social stressors that appear to have risen at the same time as the COVID-19 related deaths were divorce in Italy, pharmacy (as a marker of access to medication) in the UK, and education in the UK, Italy and Spain ( Figure 4). Abuse and divorce related searches remained stable in the UK, USA, and Spain. In Spain pharmacy related searches started to increase after lockdown was announced. In more recent days (after lockdown) there appears to be a rise in the number of Google searches for alcoholic drinks.

**Figure 5.**
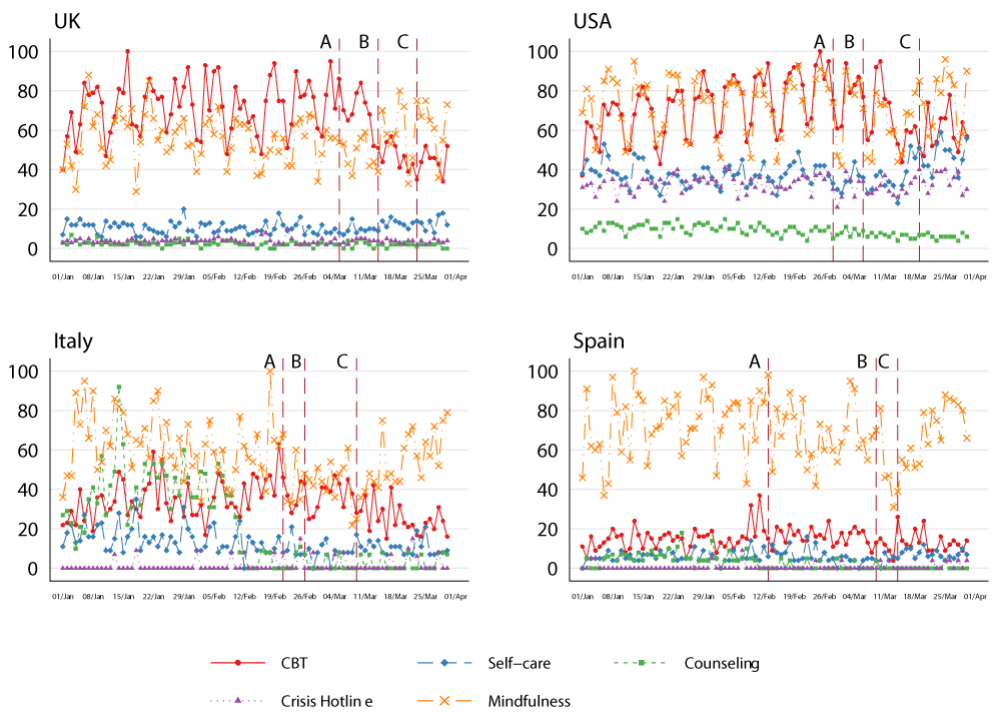
Trends in relative search volumes of key topics indicating treatment seeking in the UK, USA, Italy and Spain between 1 Jan 2020 and 30 Mar 2020. Showing figures for daily searches. A: Date of first Covid-19 related death; B: Date of first 10 deaths; C: Date of lockdown.

In the UK and Italy searches for cognitive behavioural therapy started to decline as these countries went into lockdown, potentially relating to difficulties accessing face-to-face care. Counselling searches also declined in Italy, but this decline appears to predate the first death due to COVID-19. In the USA, whilst searches for the key topics for treatment seeking remained stable, searches related to self-care started to rise after the lockdown was enforced ( Figure 5).

As a sensitivity analysis we compared the dataset downloaded and used in our main analysis with a dataset of averaged relative search values from datasets downloaded on 7 different days. The two datasets were well correlated for the majority of search topics in most countries ( Table 4), with the exception of topics in countries with low relative search values for that topic (typically below 10 - e.g. food bank in Italy and Spain (relative search values varied from 0 to 1)), and less populous countries. The use of the averaged dataset, however, did not alter our overall conclusions.

**Table 4.** Correlation between single day verses seven-day averaged Google Trend data.

	Worldwide	UK	USA	Spain	Italy
Depression	0.99	0.96	0.98	0.89	0.64
Anxiety	0.98	0.87	0.95	0.93	0.81
Suicide	0.99	0.99	0.98	0.96	0.97
Fear	0.99	0.85	0.99	1.00	0.95
Loneliness	0.97	0.61	0.68	0.35	1.00
Eviction	0.99	0.91	0.90	0.68	0.84
Welfare	0.98	0.82	0.90	0.56	0.88
Mortgage Loan	1.00	1.00	1.00	0.53	0.99
Unemployment	1.00	0.97	1.00	1.00	0.99
Food Bank	0.91	0.99	0.97	0.54	0.69
Divorce	0.96	0.93	0.98	0.80	0.90
Abuse	0.93	0.87	0.95	0.65	0.53
Alcohol	1.00	1.00	1.00	0.94	1.00
Education	1.00	0.99	1.00	1.00	0.97
Pharmacy	0.99	0.99	0.98	0.99	1.00
CBT	0.98	0.93	0.96	0.59	0.77
Selfcare	0.95	0.64	0.89	0.40	0.59
Counselling	0.99	0.48	0.85	0.86	0.93
Crisis Hotline	0.73	0.47	0.77	0.49	0.45
Mindfulness	0.97	0.91	0.95	0.77	0.73

*complete correlation between two datasets is expressed as -1 or 1

## Conclusions

Using near real-time search data from Google Trends, we show that there are individual concerns about work (unemployment) and the economic (mortgage loans and food banks) consequences of the pandemic around the world. There is an indication that relative searches related to mental distress (depression and suicide) started to fall as the number of COVID-19 deaths started to rise, with evidence that suicide related searches have started to increase after the initiation of lockdown. Searches for education and pharmacy (a marker of medication access) have started to rise. As face-to-face access to treatment declined in countries as a consequence of lockdowns, so did searches for cognitive behavioural therapy and counselling, whilst searches related to self-care increased.

The importance of considering population level psychological well-being and mental health during the COVID-19 pandemic has been recognised by the World Health Organisation ^23^. Changes in online searching behaviour assessed through Google trends has been used previously to forecast suicide occurrences in the UK and Ireland ^24, 25^. A greater number of searches for the term ‘depression’ was related to fewer suicides, whereas a greater number of searches for the term ‘suicide’ was related to more suicides which may have implications for the trends we found. However, a study in Australia did not find Google Trends to be a straightforward surveillance tool for monitoring suicide ^26^. The latter study found a lack of seasonality and only limited evidence of an association between searches related to suicide and unemployment levels. Increased Google searches related to psychological distress were associated with increases in defaulted mortgage payments, and unemployment after the 2008 recession in the USA ^27^.

Evidence from our study showing levels of mental distress are rising during the COVID-19 pandemic are consistent with emerging findings from Italy and the UK ^28– 30^. There is evidence that the impact of the public health measures are affecting different groups in different ways, with increased loneliness being reported in the elderly and higher levels of anxiety in those with poorer health ^29, 30^. Concerns related to loss of income, and practical challenges related to accessing food and shopping associated to the pandemic were also observed in other studies ^29– 31^.

In a global crisis like the COVID-19 pandemic, real time infection surveillance is a key priority in order to ensure the public health measures being introduced are effective in curbing the spread of the disease. Of equal importance is ensuring that the measures being implemented do not have unintended consequences, especially in relation to mental health and that public health messaging is tailored to evolving concerns. In Italy, Barari *et al.* (2020) recommended, based on a rapid representative survey, that communications should move from ‘stay at home’ to what the population could do while at home to ensure adherence to public health measures over time. In order to do this, real time data on individual concerns needs to be monitored, but this is difficult to do. We have shown that Google Trends could be utilised to monitor public concerns related to the pandemic. Alternative sources of data include frequently repeated (e.g. monthly) linked cross-sectional surveys of the general population, but these may be costly to implement and may provide information in a less timely fashion. In some countries (e.g. UK) there have been efforts to limit any negative impact on people’s finances through, for example, government funded financial safety nets – it is still too early to see whether these intervention strategies will have an impact on individual concerns.

Whilst there are several strengths to using this approach for monitoring individual concerns, there are limitations to this method. First, the approach requires individuals to have access to the internet and to be actively engaging in searching. This may, therefore, not represent the total population and may exclude important vulnerable groups (e.g. the elderly and those in resource poor settings). Second, there is no way of knowing who is searching for these topics. We tried to only include topics which would relate to individual concerns (e.g. unemployment versus recession). However, it has been noted previously, that Google Trends may not be valid for behavioural forecasting since we cannot ascertain who searches for certain terms and why ^21^. We were also unable to determine the sociodemographic characteristics of those conducting searches. Third, the format in which Google trends data are available for download does not allow us to estimate actual search volumes, although an indication of this can be gained by the relative stability of trend lines. In addition, trends may be affected by overall search volumes. If there is a surge in overall Google searching (e.g. during a global pandemic) this may push down the trend lines for other indicators that have remained constant in terms of search volume ^32^. Fourth, we present a descriptive analysis of Google trends data, and a statistical analysis which tests for differences in search activity before and after sentinel dates (i.e. an interrupted time-series analysis) may have strengthened our study. There were, however, at the time of this analysis limited data points during the post-outbreak period, which may have led to this type of analysis being underpowered. Since we were assessing whether it would be meaningful to use Google Trends data as a possible surveillance tool for monitoring public concerns related to the pandemic in near real-time we felt it was important to produce a timely responsive exploration of the data. Future studies, after more time has elapsed, should investigate differences in search activity using interrupted time-series. Lastly, it is unclear exactly how each Google topic is constructed and what search terms would be included. In order to ensure we used appropriate topics we checked that the most popular related queries/searches to our included topic accurately reflected the concept we were intending to investigate.

Despite these limitations, monitoring Google trends shows promise as a means of tracking changing public concerns and in weeks to come may enable policy makers to assess the impact of their interventions.

## Data availability

### Source data

All data are freely available directly from Google Trends ( https://trends.google.com/trends/)

### Underlying data

Open Science Framework: Mapping population mental health concerns related to COVID-19 and the consequences of physical distancing: a Google trends analysis. https://doi.org/10.17605/OSF.IO/UNCW2 ^22^

This project contains the following underlying data:

- averages2020FINAL.xls (Averaged dataset for sensitivity analysis)
- coviddata2020mainFINAL.xls (Combined downloaded data from Google Trends (2020))
- coviddatamainFINAL.xls (Combined downloaded data from Google Trends (2019–2020))

Data are available under the terms of the Creative Commons Zero "No rights reserved" data waiver (CC0 1.0 Public domain dedication).
