# Supplementary material for: Mapping population mental health concerns related to COVID-19 and the consequences of physical distancing: a Google trends analysis
Source: Wellcome Open Res. 2020 Jun 10;5:82. Originally published 2020 Apr 30. [Version 2] doi: 10.12688/wellcomeopenres.15870.2 (PMC7331103; doi:10.12688/wellcomeopenres.15870.2)
Supplement: Peer review history

# Peer review: Reviewer response for version 2

**DOI:** 10.21956/wellcomeopenres.17574.r39018

**Published:** 2020-07-01

**Authors:** Olivia J. Kirtley

**Affiliations:** 1 Department of Neurosciences, Center for Contextual Psychiatry, KU Leuven, Leuven, Belgium

Many thanks to the authors for their thoughtful and thorough responses to mine and the other reviewer's comments. I am satisfied that my queries regarding the manuscript have been addressed by the authors' amendments.

Is the work clearly and accurately presented and does it cite the current literature?

Yes

If applicable, is the statistical analysis and its interpretation appropriate?

Not applicable

Are all the source data underlying the results available to ensure full reproducibility?

Yes

Is the study design appropriate and is the work technically sound?

Yes

Are the conclusions drawn adequately supported by the results?

Yes

Are sufficient details of methods and analysis provided to allow replication by others?

Partly

Reviewer Expertise:

Psychology; suicide and self-harm research; theoretical models of suicidal behaviour.

I confirm that I have read this submission and believe that I have an appropriate level of expertise to confirm that it is of an acceptable scientific standard.

# Peer review: Reviewer response for version 2

**DOI:** 10.21956/wellcomeopenres.17574.r39017

**Published:** 2020-06-11

**Authors:** John Gunn

**Affiliations:** 1 Center on Gun Violence Research, Rutgers, The State University of New Jersey, Newark, NJ, USA

I am very satisfied with the adjustments to the manuscript as well as the authors responses to my suggestions. Overall I think this is a strong piece that highlights the potential for utilizing search trends as a tool for monitoring potential risk at a macro-level.

Is the work clearly and accurately presented and does it cite the current literature?

Yes

If applicable, is the statistical analysis and its interpretation appropriate?

Yes

Are all the source data underlying the results available to ensure full reproducibility?

Yes

Is the study design appropriate and is the work technically sound?

Partly

Are the conclusions drawn adequately supported by the results?

Partly

Are sufficient details of methods and analysis provided to allow replication by others?

Yes

Reviewer Expertise:

Suicide, Theories of Suicide, Risk and Protective Factors for Suicide Across the Lifespan, Impact of Media on Suicide.

I confirm that I have read this submission and believe that I have an appropriate level of expertise to confirm that it is of an acceptable scientific standard.

# Peer review: Reviewer response for version 1

**DOI:** 10.21956/wellcomeopenres.17407.r38594

**Published:** 2020-05-27

**Authors:** Olivia J. Kirtley

**Affiliations:** 1 Department of Neurosciences, Center for Contextual Psychiatry, KU Leuven, Leuven, Belgium

Thank you for the invitation to review this manuscript.

 The study utilised Google Trends data in order to provide insights into public concerns during COVID-19, across four specific countries (UK, USA, Italy and Spain), as well as worldwide. Search terms investigated fall within four areas: mental distress; economic stressors; social stressors and treatment seeking.

 I am really pleased to see underlying data being shared via the Open Science Framework. Open science practices are underused within suicide research and clinical psychology/psychiatry research more generally, so this is encouraging to see. The authors have provided a healthy critique of Google Trends data within their conclusions section and are realistic about what these data can and cannot tell us.

 I have a few comments and suggestions, which I hope will assist the authors in strengthening their manuscript.

**Methods:**

1. I would like to see some further information provided regarding how search terms were chosen and entered into Google Trends, as this was not 100% clear to me from reading the description in the paper.
2. Additionally, as Nuti *et al*. (2014) and Tran *et al*. (2017) both pointed out that different results can be obtained for the same search terms entered with and without quotation marks, it would also be useful for the authors to report in the methods section whether quotation marks were used.
  For the manuscript to be fully sound, points 1 and 2 should be addressed in the interests of replicability. 
3. Again, relating to the search terms, can the authors please explain why search terms in relevant other languages, i.e. Spanish and Italian, were not used? If I understand correctly from the methods, only English language search terms were employed. In Tran *et al.*'s (2017) study, German search terms were used when investigating Google Trends data for Germany, Switzerland and Austria. Only including English language search terms may mean that generalisability of the findings is limited. 

Is the work clearly and accurately presented and does it cite the current literature?

Yes

If applicable, is the statistical analysis and its interpretation appropriate?

Not applicable

Are all the source data underlying the results available to ensure full reproducibility?

Yes

Is the study design appropriate and is the work technically sound?

Yes

Are the conclusions drawn adequately supported by the results?

Yes

Are sufficient details of methods and analysis provided to allow replication by others?

Partly

Reviewer Expertise:

Psychology; suicide and self-harm research; theoretical models of suicidal behaviour.

I confirm that I have read this submission and believe that I have an appropriate level of expertise to confirm that it is of an acceptable scientific standard, however I have significant reservations, as outlined above.

# Response

**Published:** 2020-06-03

**Authors:** Duleeka Knipe

**Affiliations:** University of Bristol, UK

We thank the reviewer for taking the time to review our manuscript. We have responded to each point raised by the reviewer below:

1. I would like to see some further information provided regarding how search terms were chosen and entered into Google Trends, as this was not 100% clear to me from reading the description in the paper.

*Thank you for highlighting this point. We have added the following text into the methods section of the manuscript to clarify how we selected the search topics we used in our analysis:*

“ *We identified topics to be included in this analysis based on mental distress markers which we hypothesised would be associated with the pandemic, and stressors which were being discussed in the media, Government and WHO policy briefings, and preliminary review of concerns in relation to suicide* *(1)* *at the time of this analysis (March 2020). We also drew upon the experiences of the lead author (DK) who was acting as a UK community support volunteer during the outbreak, to select topics to explore under the themes of economic and social stressors. The topics explored under the treatment seeking theme were selected based on popular methods of treatment seeking.”* 

2. Additionally, as Nuti *et al*. (2014) and Tran *et al*. (2017) both pointed out that different results can be obtained for the same search terms entered with and without quotation marks, it would also be useful for the authors to report in the methods section whether quotation marks were used.

*The reviewer raises an important point regarding the use of quotation marks. In our search strategy we did not use quotation marks because we were searching with Google topics (i.e. categories that Google assigns based on search terms that have similar meaning – see table 3). The addition of quotation marks means that Google Trends will provide data on search terms rather than topics. We chose to search for topics as opposed to terms, because the former allows for the comparison of concepts that are similar across countries. We have added the following text to the methods section of the manuscript:*

*“* *Previous analyses have highlighted that different data are provided for search terms entered with and without quotation marks in Google Trends* *(1, 2)* *. As we are exploring search topics in Google Trends, as opposed to terms, the use of quotation marks is not applicable.”*

3. Again relating to the search terms, can the authors please explain why search terms in relevant other languages, i.e. Spanish and Italian, were not used? If I understand correctly from the methods, only English language search terms were employed. In Tran *et al.*'s (2017) study, German search terms were used when investigating Google Trends data for Germany, Switzerland and Austria. Only including English language search terms may mean that generalisability of the findings is limited. 

*We thank the reviewer for this comment. The search strategy used in this analysis is different to that used in previous analysis of Google Trends which employed search terms in each of the different languages of the countries included in their studies. This approach requires a detailed knowledge of each of the languages included to ensure that the concepts that are being explored are adequately captured based on the search terms used. This was part of the rationale for choosing to use Google Trend topics as opposed to search terms. Google topics are a group of search terms identified by Google to share similar meanings across languages/countries. We have added additional text to the methods section (underlined):*

*“We explored the changes in trends of key topics in Google Trends. Topics are a group of related terms that share the same concept in any language. The use of topics in Google Trends negates the need for translating search terms.”*

# Peer review: Reviewer response for version 1

**DOI:** 10.21956/wellcomeopenres.17407.r38590

**Published:** 2020-05-12

**Authors:** John Gunn

**Affiliations:** 1 Center on Gun Violence Research, Rutgers, The State University of New Jersey, Newark, NJ, USA

This is a timely and important work that explores the trends in google searches related to mental health in the wake of the COVID-19 pandemic. While the work is valuable, there are two main points I would like to see the authors address:

1. While the authors acknowledge the flaws in google/trends analyses that are brought out from Tran *et al*. (2017) – they do not address the lack of reliability in google/trends data. As indicated, data was collected on a single day, 2 April 2020. However, google/trends data scores vary day-to-day. In order to overcome this shortcoming, Tran *et al*. (2017) recommend collecting trend data each day of a 7-day period. A final score can be computed by average across these 7-days, thus improving upon the poor reliability.
2. I would also be curious what the rationale was for providing descriptions of the trends, but exploring them statistically? Much of the google/trends work I’ve seen compares a time point prior to and following an event to determine if there are statistically significant differences. For example, do the searches in the weeks prior to lockdown differ significantly from the searches in the weeks following? Do these months of 2020 differ significantly from the same period in time in 2019, 2018, 2017,…?  I think this would add to the strength of this piece – or in the least should be discussed as a limitation with a rationale provided.

The above concerns are what drove my responses of “partly” to the questions of whether the study design is appropriate and technically sound and whether or not the conclusions drawn are adequately supported by the results. For this manuscript to be sound, I would expect item 1 to be addressed. While I think it would be useful for the authors to provide a statistical analysis per point 2, acknowledgment of this limitation and a rationale for why they did a descriptive analysis would suffice.

Is the work clearly and accurately presented and does it cite the current literature?

Yes

If applicable, is the statistical analysis and its interpretation appropriate?

Yes

Are all the source data underlying the results available to ensure full reproducibility?

Yes

Is the study design appropriate and is the work technically sound?

Partly

Are the conclusions drawn adequately supported by the results?

Partly

Are sufficient details of methods and analysis provided to allow replication by others?

Yes

Reviewer Expertise:

Suicide, Theories of Suicide, Risk and Protective Factors for Suicide Across the Lifespan, Impact of Media on Suicide.

I confirm that I have read this submission and believe that I have an appropriate level of expertise to confirm that it is of an acceptable scientific standard, however I have significant reservations, as outlined above.

# Response

**Published:** 2020-06-03

**Authors:** Duleeka Knipe

**Affiliations:** University of Bristol, UK

We thank the reviewer for taking the time to review our manuscript. We have responded to each point raised by the reviewer below:

1. While the authors acknowledge the flaws in google/trends analyses that are brought out from Tran *et al*. (2017) – they do not address the lack of reliability in google/trends data. As indicated, data was collected on a single day, 2 April 2020. However, google/trends data scores vary day-to-day. In order to overcome this shortcoming, Tran *et al*. (2017) recommend collecting trend data each day of a 7-day period. A final score can be computed by average across these 7-days, thus improving upon the poor reliability.

*We thank the reviewer for this valuable point. Tran et al (2017) do highlight in their paper that research should ideally use averaged relative search values. However, they also state that the “slightly” different search values retrieved on different days were more apparent for earlier years than latter years. In addition, they found the differences to be a particular problem for small countries (i.e. Austria/Switzerland, population size ~9 million). Our analysis is based on data from populous countries (populations >50 million) and includes data points from the start of 2020 so it is likely the reliability issues highlighted in the paper will not be applicable to our analysis. However to reassure the reviewer and ourselves, we conducted the data download of the data used in this analysis over a 7 day period and compared the 7-day averaged daily relative search values (RSVs) with those used in our original analysis. We find that for the majority of search topics there was a strong correlation between the two datasets. For topics where there were low search volumes and therefore low daily RSVs (generally less than 10), the two datasets were less strongly correlated. We have added the following text to the manuscript:*

*METHODS*

*“Previous analysis of Google Trends data has highlighted that slightly different relative search values are provided by Google for the same search (with the same parameters) on different days* *(3)* *. This has been highlighted to be a problem when data for earlier years are downloaded and for smaller countries. As a sensitivity analysis we generated an averaged dataset for the topics included in this analysis. The averaged dataset was created by re-running the same Google Trends search query with the same parameters for time period (i.e. 1 Jan 2020 – 30 Mar 2020), location (e.g. USA), and each set of topics (e.g. mental distress topics) on 7 different days.* *We took the average value for each search topic (20 topics) on each of the 90 data points for all 5 settings, as it was recorded on each of the 7 separate days. We then correlated that 7-day average with values for the 90 days (1 Jan 2020- 30 Mar 2020) used in the main analysis (which used values as they stood when the data were extracted on 2 Apr 2020). We checked to see whether the use of the averaged dataset would have altered our overall conclusions.”*

*RESULTS*

*"As a sensitivity analysis we compared the dataset downloaded and used in our main analysis with a dataset of averaged relative search values from datasets downloaded on 7 different days. The two datasets were well correlated for the majority of search topics in most countries (Table 4), with the exception of topics in countries with low relative search values for that topic (typically below 10 -* *e.g. food bank in Italy and Spain (relative search values varied from 0 to 1)), and less populous countries. The use of the averaged dataset, however, did not alter our overall conclusions. "*

2. I would also be curious what the rationale was for providing descriptions of the trends, but exploring them statistically? Much of the google/trends work I’ve seen compares a time point prior to and following an event to determine if there are statistically significant differences. For example, do the searches in the weeks prior to lockdown differ significantly from the searches in the weeks following? Do these months of 2020 differ significantly from the same period in time in 2019, 2018, 2017,…?  I think this would add to the strength of this piece – or in the least should be discussed as a limitation with a rationale provided.

*The reviewer raises an important point regarding our analysis strategy. One of the reasons for conducting this study was to see whether it would be meaningful to use Google Trends data as a possible surveillance tool for monitoring public concerns related to the pandemic in near real-time. Based on this we were keen to provide a first look at the data quickly in response to the crisis. We discussed the possibility of conducting an interrupted time-series analysis but felt that there would not have been sufficient data points during the ‘after’ period to conduct a meaningful analysis. We have added the following text to the limitations of our manuscript:*

*“* *Fourth, we present a descriptive analysis of Google trends data, and a statistical analysis which tests for differences in search activity before and after sentinel dates (i.e. an interrupted time-series analysis) may have strengthened our study. There were, however, at the time of this analysis limited data points during the post-outbreak period, which may have led to this type of analysis being underpowered. Since we were assessing whether it would be meaningful to use Google Trends data as a possible surveillance tool for monitoring public concerns related to the pandemic in near real-time we felt it was important to produce a timely responsive exploration of the data. Future studies, after more time has elapsed,* *should investigate differences in search activity using interrupted time-series.* *”*
